# Should rescue ICSI be re-evaluated considering the deferred transfer of cryopreserved embryos in in-vitro fertilization cycles? A systematic review and meta-analysis

**DOI:** 10.1186/s12958-021-00784-3

**Published:** 2021-08-04

**Authors:** Alessio Paffoni, Marco Reschini, Valerio Pisaturo, Cristina Guarneri, Simone Palini, Paola Viganò

**Affiliations:** 1 Infertility Unit, ASST Lariana, Cantù, Como, 22063 Italy; 2 Infertility Unit, Fondazione IRCCS Ca’ Granda Ospedale Maggiore Policlinico, Milan, 20122 Italy; 3 IVF Unit, AUSL Romagna Cervesi Hospital, 47841 Cattolica, Italy

**Keywords:** IVF, Total fertilization failure, Rescue ICSI, Delayed ICSI, Intracytoplasmic sperm injection

## Abstract

**Background:**

Total fertilization failure represents a particularly frustrating condition for couples undergoing in vitro fertilization. With the aim of reducing the occurrence of total fertilization failure, intracytoplasmic sperm injection (ICSI) has become the first choice over conventional in vitro fertilization (IVF) procedures although evidence of improved results is still debated and its use in couples without male factor infertility is not recommended. Among the strategies potentially useful to promote the use of conventional IVF, we herein call attention to the late rescue ICSI, which consists in performing ICSI after 18–24 h from conventional insemination on oocytes that show no signs of fertilization. This treatment has however been reported to be associated with a low success rate until recent observations that embryos derived from late rescue ICSI may be transferred after cryopreservation in a frozen-thawed cycle with improved results. The aim of the present study was to assess whether frozen embryos deriving from rescue ICSI performed about 24 h after conventional IVF may represent a valuable option for couples experiencing fertilization failure.

**Methods:**

A systematic review on the efficacy of late rescue ICSI was performed consulting PUBMED and EMBASE.

**Results:**

Including twenty-two original studies, we showed that clinical pregnancy rate per embryo transfer and implantation rate obtainable with fresh embryo transfers after rescue ICSI are not satisfactory being equal to 10 and 5%, respectively. The transfer of cryopreserved rescue ICSI embryos seems to offer a substantial improvement of success rates, with pregnancy rate per embryo transfer and implantation rate equal to 36 and 18%, respectively. Coupling rescue ICSI with frozen embryo transfer may ameliorate the clinical pregnancy rate for embryo transfer with an Odds Ratio = 4.7 (95% CI:2.6–8.6).

**Conclusion:**

Results of the present review support the idea that r-ICSI coupled with frozen embryo transfer may overcome most of the technical and biological issues associated with fresh transfer after late r-ICSI, thus possibly representing an efficient procedure for couples experiencing fertilization failure following conventional IVF cycles.

**Trial registration:**

Prospero registration ID: CRD42021239026.

**Supplementary Information:**

The online version contains supplementary material available at 10.1186/s12958-021-00784-3.

## Background

Intracytoplasmic sperm injection (ICSI), initially developed to treat severe male infertility, was introduced in the early 1990s as one of the most dramatic technological breakthroughs in assisted reproductive technology (ART) [1]. The technique was rapidly integrated into the routine clinical practice and is presently considered the most widely used insemination method worldwide [2]. The reliability in achieving fertilization in cases of severe male factor infertility has led to the expansion of its use also for other infertility indications. In the United States, ICSI use increased from 36% in 1996 to 76% in 2012, with the largest relative increase among ART cycles without an indication of male factor infertility. According to Zagadailov et al. [3], state mandates for ART coverage can encourage more restrictive use of laboratory resources. From 2000 to 2016, absolute rates of ICSI use per clinic increased by 20% in both ART-mandated (42.5 to 62.5%) and non-mandated states (46.9 to 67.6%) with statistically significant lower ICSI utilization in insurance-mandate states. An increase in ICSI use has been reported in several countries worldwide, with ICSI rate close to 100% in the Middle East [4].

Reducing the occurrence of total fertilization failure (TFF) represents the plausible reason for this “indication creep” of ICSI over conventional in vitro fertilization (IVF) procedures. Total fertilization failure represents a particularly frustrating condition for couples undergoing ART and for professionals since it results in the premature termination of the cycle. Its incidence following conventional insemination is not infrequent, being estimated to range between 5 and 20% [5]. Notably, however, evidence of improved fertilization results with the use of ICSI is still debated and strongly related to the infertility indication considered [6–9]. Furthermore, clues in support for the need to limit widespread use of ICSI stem from inconclusive data on improved post-fertilization reproductive outcomes for non-male factor infertility diagnosis and from the significantly higher rate of de novo, chromosomal abnormalities and birth defects observed in children born after ICSI compared with the rate in the general population [10]. It is therefore, not unexpected that the Practice Committee of the American Society for Reproductive Medicine and the Society for Assisted Reproductive Technology, have declared that there is insufficient evidence to suggest ICSI use in couples without male factor infertility [10].

Among the strategies potentially useful to promote the use of conventional IVF, we herein call attention to the rescue ICSI (r-ICSI), which consists in performing ICSI after 4–24 h from conventional insemination on oocytes that show no signs of fertilization. This treatment, potentially valuable in rescuing cycles with total or partial fertilization failure, has however been reported to be associated with a low success rate when performed after 24 h (late r-ICSI) [11]. Reasons underlying this low rate may include the time-dependent deterioration in oocyte quality and the loss of synchronization between endometrial growth and embryo development. To limit these deleterious effects, a r-ICSI strategy to be carried out approximately 4–8 h after conventional insemination (early r-ICSI) has been proposed, allowing to obtain higher fertilization rates [12]. Unfortunately, given its difficult implementation, poorly compatible with the organization of a laboratory, the technique is currently quite unpopular [11, 13].

Recently, a step forward in this context has derived from the observation that embryos derived from late r-ICSI may be transferred after cryopreservation in a frozen-thawed cycle with improved results [14, 15]. The strategy of cryopreservation could overcome all the technical and biological issues associated with late r-ICSI, allowing the procedure to be more frequently used in limiting the risk of TFF associated with conventional IVF cycles. Therefore, in the present systematic review, we sought to verify whether r-ICSI coupled with frozen embryo transfer may favor ART success rate of couples experiencing TFF following conventional IVF cycles.

## Methods

Studies were considered for inclusion in the systematic review following the PICOC framework as follows - patients/population: couples undergoing IVF cycles; intervention: rescue (delayed) ICSI performed on the day after oocyte retrieval and TFF following conventional IVF; comparison: when possible, r-ICSI coupled with frozen embryo transfer compared to r-ICSI with fresh embryo transfer; main Outcome: clinical pregnancy rate per cycle (clinical evidence of intrauterine foetal sac); additional outcomes: fertilization rate, implantation rate, ongoing pregnancy rate, delivery rate, malformation rate according to the International Classification of Diseases 11th Revision [16]; clinical outcomes were calculated separately for fresh and frozen embryo transfers; context: r-ICSI has been reported to be associated with a low efficacy and this may be explained by the asynchrony between embryo development and endometrial receptivity.

The following search string was used in PUBMED and EMBASE on 23^rd^ February 2021 and repeated on 19^th^ April 2021:

((((“rescue ICSI”) OR (R-ICSI)) OR (“rescue intracytoplasmic sperm injection”)) OR (“delayed ICSI”)) OR (“delayed intracytoplasmic sperm injection”). No restrictions were used at this stage, with the exception of the “article” publication type for EMBASE.

Two people independently screened records for inclusion and their decision was blinded to each other. A third author checked for disagreement between results and a decision was taken by three authors. This process was recorded through an excel spreadsheet. Reference lists cited in study reports included in the systematic review were examined in order to retrieve additional papers suitable for inclusion.

For data extraction, studies were included in the data synthesis if reporting: 1) clinical pregnancy rate per cycle after r-ICSI performed on the day after oocyte retrieval; 2) indication regarding fresh or frozen embryo transfer; 3) results published in full in English language. The following data were extracted: First Author, publication ID, year of publication, period of recruitment, study design, mean age of included women, inclusion of cases with total or partial fertilization failure, fertilization rate with conventional IVF, number of cycles included, time of r-ICSI after conventional insemination, use of sperm from previous day or freshly collected, number of oocytes treated with r-ICSI, r-ICSI fertilization rate and abnormal fertilization rate, number of embryos obtained, number of fresh embryos transferred, number of transferred frozen embryos, technique of cryopreservation, strategy of endometrial preparation, number of pregnancies, number of newborns, number of newborns with malformations. Those data were recorded in an excel spreadsheet and were used to calculate the main outcomes and to account for possible heterogeneity among studies. Studies including a comparison between r-ICSI cycles with fresh and frozen embryo transfer were also considered for quantitative evaluation. If not clearly indicated, the number of inseminated or fertilized oocytes and the number of transferred or obtained embryos were calculated using available data such as mean values or rates. Two reviewers collected data from each report working independently; disagreements between data collectors were resolved with the intervention of a third reviewer and collegial discussion.

Quality assessment of included papers was performed using the “JBI critical appraisal checklist for cross sectional studies” [17], an evaluation tool developed to evaluate representativeness and reliability of studies. Each of 8 criteria was assessed (Yes, No, Unclear, Not applicable) by two independent reviewers and disagreements were solved in a collegial discussion with a third reviewer after reconsidering the following aspects: criteria for inclusion in the sample, description of basal characteristics of patients/cycles, methodological definition of r-ICSI, identification of confounding factors and strategies to deal with them, definition of the outcomes including pregnancy rate, use of statistical analysis.

A narrative and tabular synthesis was used for presenting the outcomes. Confidence intervals of proportions for the narrative synthesis were obtained with a binomial exact calculation. Clinical outcomes (fertilization rate, pregnancy rate per embryo transfer/cycle, implantation rate) were synthesised with the inverse-variance method. Odds ratios (OR) were obtained for case/control studies comparing fresh and frozen cycles using the Mantel-Haenszel method. A visual synthesis of results was obtained with forest plots. Analysis and figures were done with R packages [18]. Studies with missing values were excluded from the synthesis of the specific outcome. With the main goal to estimate the mean effect in a range of studies, a random-effect model was selected to conduct the meta-analysis; in case of low inconsistency (I^2^ < 30%), results obtained with a fixed model were also included. τ^2^ was reported as a measure of heterogeneity among studies.

## Results

**Fig. 1:**
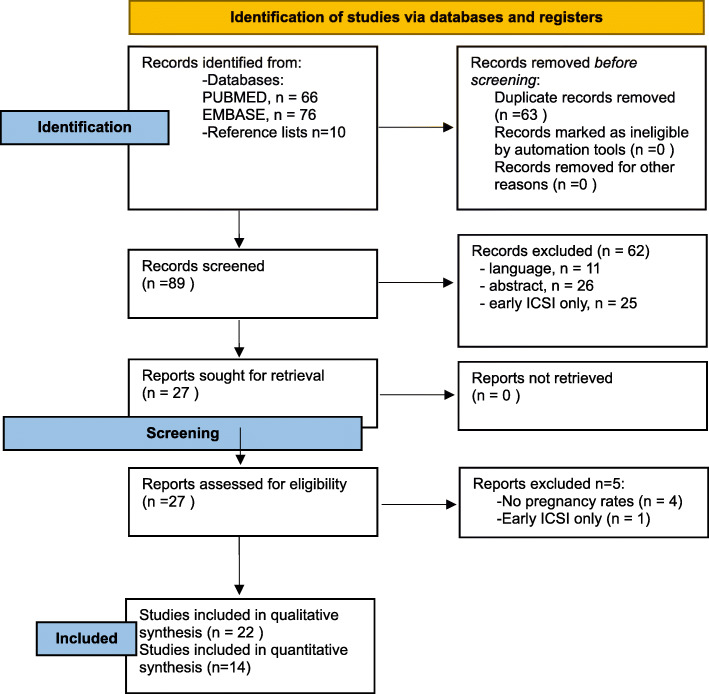
Prisma 2020 flow-diagram of study selection process

Studies were identified and selected for inclusion in the review as reported in the flowchart (Fig. 1). Out of 89 initially retrieved studies through PUBMED/EMBASE search and reference lists, 22 were finally included [12, 14, 15, 19–37]. The key characteristics and results of the studies are summarized in Table 1. Eight case-reports were used for qualitative synthesis of data regarding late r-ICSI but were excluded from pregnancy and implantation rates calculation [21, 25–27, 29, 30, 34, 35]. Fourteen retrospective studies were included in the quantitative synthesis [12, 14, 15, 19, 20, 22–24, 28, 31–33, 36, 37] and three studies were also used to calculate the OR for pregnancy and implantation rates between frozen and fresh embryo transfer after r-ICSI [14, 15, 22]. Main results of selected studies including transfer of fresh and frozen rescue ICSI embryos are reported in Tables 2 and 3, respectively. An additional table shows the critical appraisal of included studies according to the Joanna Briggs Institute checklist (see Additional file 1).

**Table 1:** Characteristics of included studies

First Author, year [ID]	Country	Years recruitment	Source	Type of study	total (TFF) or partial (PFF) fertilization failure	total number of IVF cycles	female age mean (±SD)	n° of r-ICSI cycles	n° of r-ICSI oocytes	timing of r-ICSI post IVF (h)	Sperm
Lundin K, 1996 [19]	Sweden	< 1995	Ref	R	TFF and PFF			57	450	20–22	P,F
Morton PC, 1997 [20]	Usa	1993–1996	Ref	R	TFF		35.3 ± 4.3	54	489	20–24	P
Bussen, 1997 [21]	Germany	< 1997	Ref	CR	PFF		32	1	6	20	P
Yuzpe AA, 2000 [22]	Canada	1997–1999	Pub/Emb	R	TFF and PFF	535	34.4 ± 4.0	32	234	19–22	P
Park KS, 2000 [23]	Korea	< 2000	Ref	R	TFF and PFF		31.7 ± 1.6	17	68	> 18	P
Kuczyński W, 2002 [24]	Poland	1996–2000	Pub/Emb	R	TFF	1412	32.9 ± 5.0	120	779	18–20	
Chian RC, 2003 [25]	Canada	< 2003	Pub/Emb	CR	TFF		29	1	4	> 18	P
Lombardi E, 2003 [26]	Argentina	1998	Pub/Emb	CR	PFF		36	1	12	20	P
Chen C, 2003 [12]	Singapore	1997–1998	Pub/Emb	R	TFF	230	35.2 ± 4.1	20	182	22	
Pehlivan T, 2004 [27]	Spain	< 2003	Pub/Emb	CR	PFF		35	1	11	21	
Amarin ZO, 2005 [28]	Saudi Arabia	1995–2001	Pub/Emb	R	TFF	492	32.6	78	616	> 18–24	P
DeUgarte CM, 2006 [29]	Usa	< 2005	Pub/Emb	CR	TFF		42	1	7	20	P
Esfandiari N, 2008 [30]	Canada	2007	Pub/Emb	CR	PFF		28	1	8	19	
Sermondade N, 2010 [15]	France	2004–2009	Pub/Emb	R	TFF		35,5 ± 3.6	17	127	> 18	P
Shalom-paz E, 2011 [31]	Canada	1999–2008	Pub/Emb	R	TFF	2700	35,5 ± 4,5	92	883	16–18	P
Xiong S, 2011 [32]	China	2009	Pub/Emb	R	TFF		35.3 ± 3.2	3	20	20	P
Zhu L, 2011 [33]	China	2007–2009	Emb	R	TFF		31.3 ± 5.3	16	98	20–22	P
Ming L, 2012 [14]	China	2006–2011	Pub/Emb	R	TFF	15,162	31.1–33.2 ± 4.3	534	4824	> 16–18	P
Singh N, 2013 [34]	India	< 2010	Pub/Emb	CR	TFF		32	1	4	> 18	P
Moon JH, 2015 [35]	Canada	< 2014	Pub/Emb	CR	TFF		28.5	2	19	21	
Sachdev NM, 2016 [36]	Usa	2003–2015	Pub/Emb	R	TFF and PFF		36,7 ± 4,3	12	111	15–18	
Li M, 2021 [37]	China	2013–2016	Pub/Emb	R	TFF	27,582	31.4–32.4 ± 4.7	625	3993	> 16–19	P

Empty cells = missing values; *r-ICSI* rescue-ICSI; *Pub* Pubmed; *Emb* Embase, *Ref* cited references; *R* retrospective/cross-sectional; *CR* case-report; *P* previous day; *F* fresh

**Table 2:** Main results of selected studies including transfer of fresh rescue ICSI embryos

First Author, year [ID]	2PN(%)	n° patients	Patients without viable embryos	n° ET	n° of obtained embryos	n° of fresh embryos transferred	developmental stage	n° clinical pregnancies	n° ongoing pregnancies or deliveries	n° embryos impanted	n° malformations/newborns
Lundin K, 1996 [19]	46.9	48	18	29		57	cleavage	2	2	2	0/2
Morton PC, 1997 [20]	44.0	54	5	48	164	140	cleavage	8	8	15	0/15
Bussen, 1997 [21]	50.0	1	0	1		2	cleavage	1	1	1	0
Yuzpe AA, 2000 [22]	60.2	32	3	27		89	cleavage	5	3	6	0/4
Park KS, 2000 [23]	47.1	17		1	4	4		0	0	0	
Kuczyński W, 2002 [24]	30.4	120		100		166	cleavage	0	0	0	
Chian RC, 2003 [25]	100	1	0	1	4	3	cleavage	1	1	2	0
Chen C, 2003 [12]	91.7	20	0	20	73	58	cleavage	1	1	1	0/1
Pehlivan T, 2004 [27]	36.4	1	1	0	4 (a)	0		0	0	0	
Amarin ZO, 2005 [28]	50.9	78	14	64	208	174	cleavage	4	3	4	0/3
DeUgarte CM, 2006 [29]	42.9	1	0	1	3	1	blastocyst	1	1	1	0
Esfandiari N, 2008 [30]	87.5	1	0	1	6	6	cleavage	1	1	3	
Sermondade N, 2010 [15]	60.9	17		15	55	35	cleavage	1	1	1	
Shalom-paz E, 2011 [31]	56.2	92	0	92		278	cleavage	15	10	17	0/12 (b)
Xiong S, 2011 [32]	87.7	3	1	2		3	cleavage	0	0	0	
Zhu L, 2011 [33]	52.0	16	4	12	21	20	cleavage	0	0	0	
Ming L, 2012 [14]	45.0	534	62	469	1416	1000	cleavage	40	31	45	0/16 (c)
Singh N, 2013 [34]	75	1	0	1	3	3	cleavage	1	1	1	0
Moon JH, 2015 [35]	63.2	2	0	2	7	2	blastocyst	2	1	2	0

Empty cells = missing values; 2PN = 2 pronuclei; ET = Embryo Transfer

a) no suitable embryos after genetic screening for aneuploidies; b) + 1 termination for Down syndrome; c) + 1 termination for eye defect

**Table 3:** Main results of selected studies including transfer of cryopreserved rescue-ICSI embryos

First Author, year [ID]	embryo cryopreservation, indication	technique of cryopreservation	n° of patients	endometrial preparation	n° ET	n° of frozen embryos transferred	developmental stage	n° embryos implanted	n° clinical pregnancies	n° ongoing pregnancies or deliveries	n° malformations/newborns
Yuzpe AA, 2000 [22]	Supernumerary	Slow freezing	2	P	2	7	cleavage	1	1	1	0/1
Lombardi E, 2003 [26]	Supernumerary	Slow freezing	1	P	1	5	cleavage	1	1	1	0
Sermondade N, 2010 [15]	Supernumerary	Slow freezing	5		5	12	cleavage	2	2	1	0/1
Ming L, 2012 [14]	Supernumerary	Slow freezing	64	N/P	64	165	cleavage	22	19	15	0/12
Moon JH, 2015 [35]	Supernumerary	Vitrification	1	P	1	1	blastocyst	1	1	1	0
Sachdev NM, 2016 [36]	Elective PGT	Vitrification	12	N/P	3	3	blastocyst	3	3	3	0/3
Li M, 2021 [37]	Elective	Vitrification	332	N/P	332	594	cleavage/ blastocyst	141	122	99	1/109

*Empty cells* missing values; *ET* embryo transfer; *N* Natural; *P* Programmed, *PGT* pre implantation genetic testing

As reported in Table 1, a total of *n* = 1686 late r-ICSI cycles with *n* = 12,945 inseminated oocytes were reported in 22 studies. Rescue ICSI was performed after 15–24 h from initial conventional IVF. The number of clinical pregnancies following r-ICSI were reported to be *n* = 83 in fresh cycles and *n* = 149 in frozen cycles with *n* = 65 and *n* = 121 ongoing pregnancies/deliveries, respectively. The rate of r-ICSI on the total of conventional IVF cycles was available in 7 retrospective studies [12, 14, 22, 24, 28, 31, 37] and was equal to 3.1% (95%CI: 3.0–3.3%).

In the eight case-reports, a total of nine r-ICSI cycles performed > 18 h after the conventional IVF insemination using *n* = 71 oocytes was reported. Age of included women ranged between 28 and 42 years. The cumulative normal fertilization rate was 63.4% (95%CI: 52–74%). Eighteen fresh embryos were replaced in seven embryo transfers between day 3 and 6 after oocyte retrieval; ten embryos implanted in *n* = 6 patients with an implantation rate equal to 56% (95%CI: 34–75%). In one case-report study, embryo transfer was not performed as the cytogenetic analysis demonstrated the absence of euploid embryos [27]. The delivery of at least one baby was reported in five studies [21, 25, 29, 34, 35]; the newborns were healthy and no malformations were reported. Two studies reported two successful frozen embryo transfers; one of them was performed with *n* = 4 slow-frozen embryos [26] and the other with one vitrified embryo [35]. Both pregnancies resulted in the delivery of a healthy baby.

**Fig. 2:**
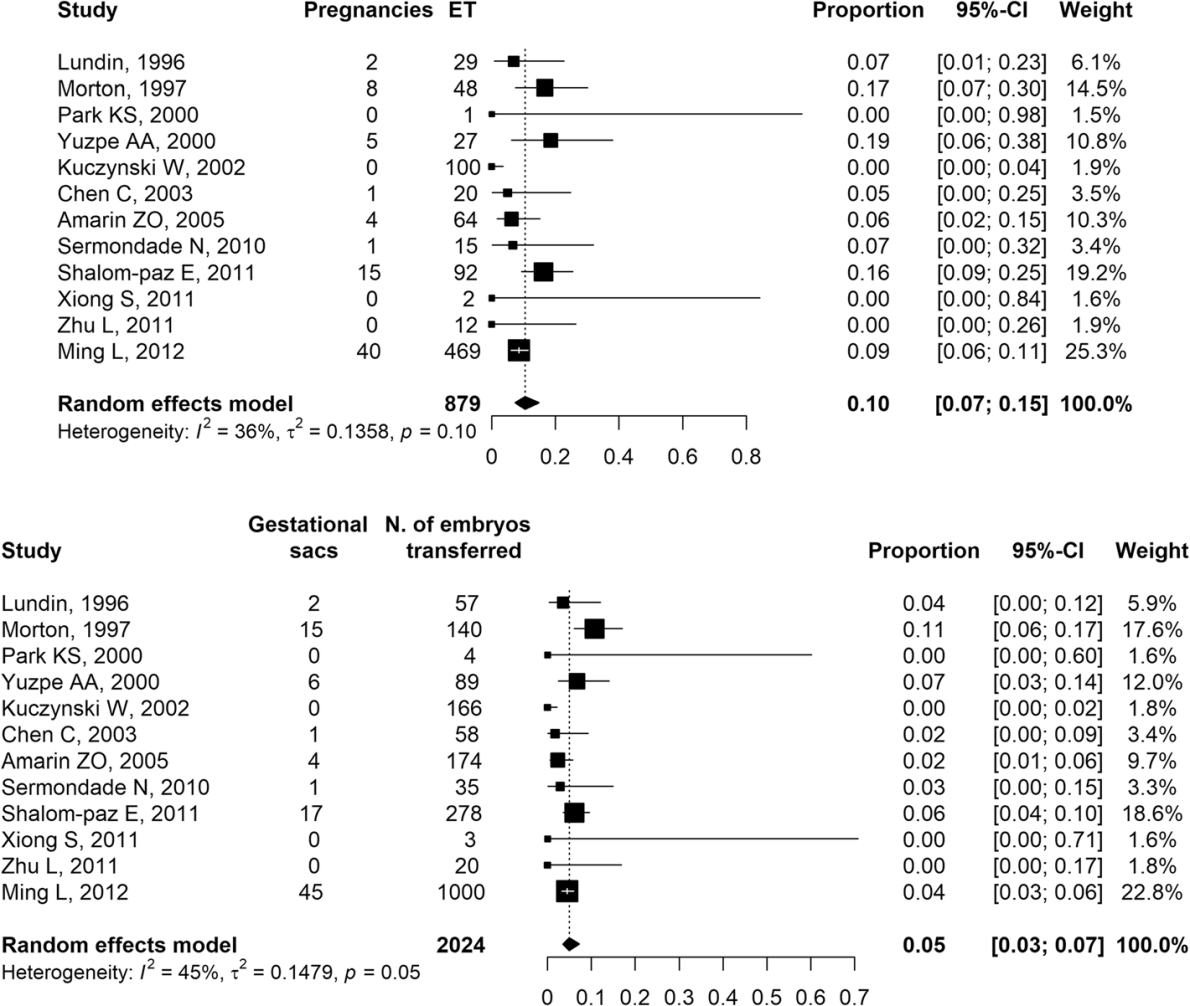
Quantitative synthesis of the studies reporting clinical pregnancy (*upper panel*) and implantation rate (*lower panel*) per embryo transfer in fresh cycles following rescue-ICSI performed 15–24 h after conventional IVF resulting in total or partial fertilization failure

In the 14 retrospective cohort studies, a total of *n* = 1677 r-ICSI cycles (range 3–625 cycles per study), performed in women with a mean age ranging from 31.1 to 36.7 years, were included [12, 14, 15, 20, 22–24, 28, 31, 33–37]. Rescue cycles were performed using *n* = 12,874 unfertilized oocytes (range 20–4824 per study) 15–24 h after conventional IVF resulting in total or partial fertilization failure. Normal fertilization rate (2 *pronuclei*) in individual studies ranged between 30 and 92% with a cumulative effect size equal to 54% (95%CI: 48–60%; I^2^ = 95%, τ^2^ = 0.17) estimated on a total of *n* = 8881 r-ICSI oocytes (the forest plot of fertilization rate is available in the Additional file 2). The rate of abnormal fertilization (1 or 3 *pronuclei*) was reported in five studies [12, 15, 19, 20, 23] and ranged between 5 and 9% of r-ICSI oocytes. In the majority of the studies, r-ICSI was performed using the sperm sample collected on the previous day; one study found a higher rate of normally fertilized oocytes using freshly collected sperm cells (51%) compared to 1-day-old-spermatozoa (36%) [18]. Twelve studies reported a total of *n* = 1031 women undergoing *n* = 879 fresh embryo transfers on day 3 or 4 after oocyte retrieval with *n* = 2024 embryos [12, 14, 15, 20, 22–24, 28, 31–33, 36]; the implantation rate ranged between 0 and 11% with an effect size equal to 5% (95%CI: 3–7%). Seventy-six clinical pregnancies were obtained corresponding to a clinical pregnancy rate per r-ICSI cycle ranging between 0 and 17% and an overall effect size equal to 10% (95%CI: 7–15%). A quantitative synthesis of implantation and clinical pregnancy rates per embryo transfer in fresh cycles is summarized in Fig. 2.

Fifty-nine ongoing/deliveries pregnancies were reported. Among fifty-three newborns from fresh transferred r-ICSI embryos, no malformations were reported; two terminations for trisomy 21 (ICD-11: LD40.0) and congenital eye abnormality (ICD-11: LA10) were recorded [14, 31]. As reported in Table 2, viable embryos can not be obtained with late r-ICSI in a proportion of women up to 38%; the crude incidence of cases without viable embryos in retrospective studies was 14% (95%CI:11–16%; *n* = 107/785).

Five studies provided results on the transfer of cryopreserved embryos following r-ICSI; in three studies, supernumerary embryos were slow-freezed after fresh embryo transfer [14, 15, 22] and in the remaining two studies, cryopreservation was elective and performed with vitrification [36, 37]. The cumulative percentage of cycles with cryopreservation of supernumerary embryos was *n* = 415/1220 (34%, 95%CI:32–37%). In the study with elective embryo vitrification coupled with preimplantation genetic testing (PGT) [36], the rate of patients receiving euploid embryos was *n* = 3/12 (25%, 95%CI:9–53%); in the second study with elective embryo vitrification, the rate of patients obtaining viable embryos was *n* = 406/625 (65%, 95%CI: 61–69%).

**Fig. 3:**
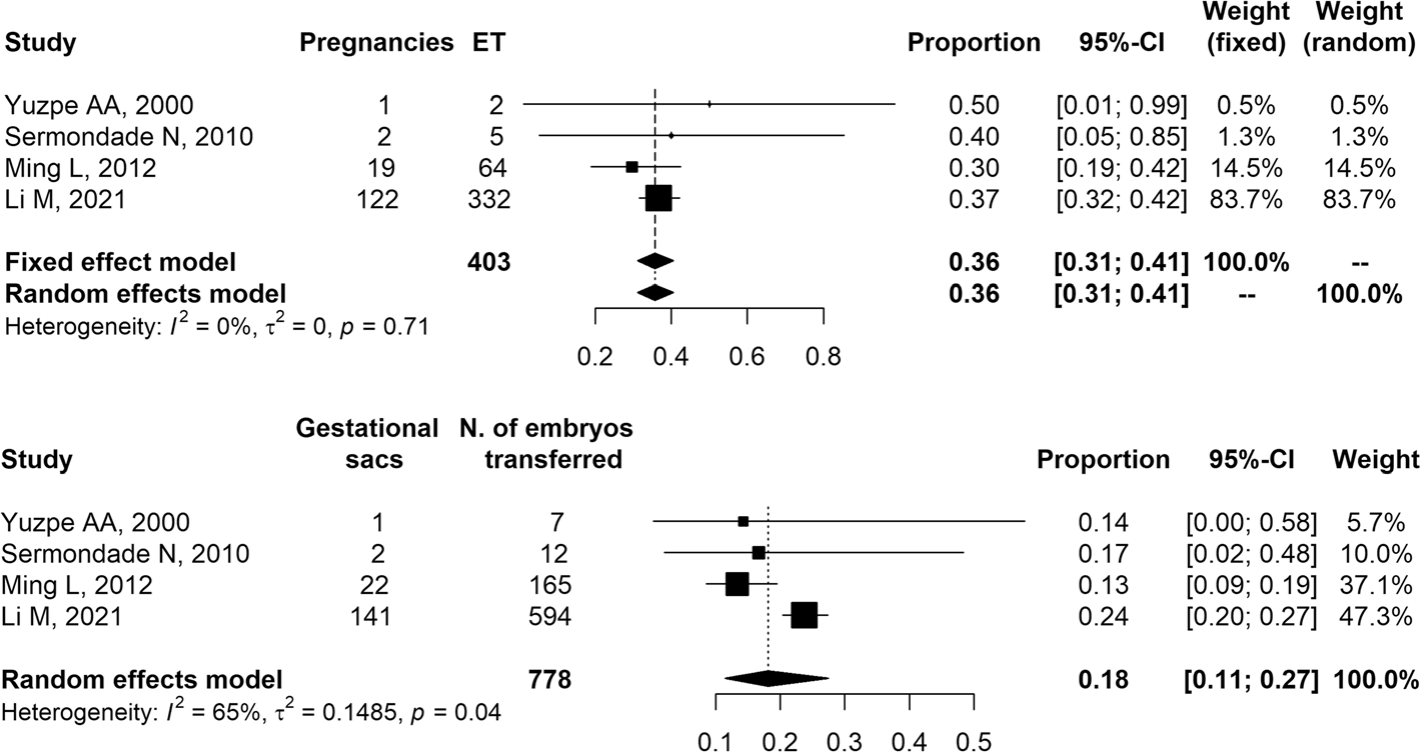
Quantitative synthesis of the studies reporting clinical pregnancy (*upper panel*) and implantation rate (*lower panel*) per embryo transfer in frozen cycles following rescue-ICSI performed 15–24 h after conventional IVF resulting in total or partial fertilization failure. Cases with preimplantation genetic testing were excluded

A total of *n* = 406 embryo transfers with frozen/thawed embryos were performed in a cohort of *n* = 415 patients. The number of embryos transferred was *n* = 781 (mean number of embryos per transfer = 1.9) and the implantation rate, excluding the study with PGT showing 100% implantation rate with 3 transferred embryos [36], ranged between 13.3 and 23.7%. The clinical pregnancy rate per started frozen cycle ranged between 25.0 and 50.0%. The quantitative synthesis showed an implantation rate equal to 18% (95%CI: 11–27%) and a pregnancy rate per embryo transfer equal to 36% (95%CI: 31–41%) following r-ICSI frozen cycles (Fig. 3). When considering only frozen embryos transferred at the cleavage stage, implantation rate was 12% (95%CI: 10–15%, I^2^ = 0%, 흉^2^ = 0).

Among *n* = 126 newborns from cryopreserved r-ICSI embryos, a case of microtia (ICD-11: LA22.0) was reported [37].

**Fig. 4:**
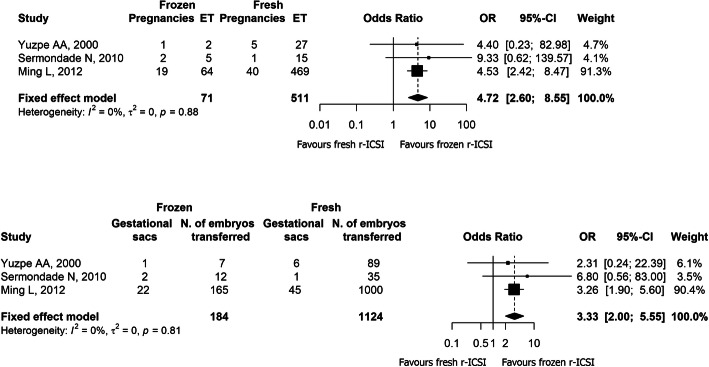
Meta-analysis of studies comparing frozen and fresh rescue-ICSI cycles. Odds Ratios (OR) for clinical pregnancy rate per embryo transfer *(upper panel)* and implantation rate *(lower panel)* are shown

In three studies, a comparison between the transfer of fresh and supernumerary frozen r-ICSI cycles was performed [14, 15, 22]. As depicted in Fig. 4, the ORs were 3.3 (95%CI: 2.0–5.5) and 4.7 (95%CI: 2.6–8.5) for implantation and clinical pregnancy rate per embryo transfer, respectively, favouring frozen embryo transfer.

## Discussion

The present review has revealed some important aspects regarding ICSI performed on the day following oocyte retrieval as a rescue procedure for fertilization failure following conventional IVF cycles: 1) the clinical pregnancy rate per embryo transfer and implantation rate obtainable with fresh embryo transfers are in general not satisfactory being equal to 10 and 5%, respectively and up to 1 out of 7 women can not obtain viable embryos despite the use of late r-ICSI; 2) the transfer of cryopreserved r-ICSI embryos seems to offer a substantial improvement of success rates, with pregnancy rate per embryo transfer and implantation rate equal to 37 and 20%, respectively. The low rates of success associated with the r-ICSI after fresh transfers have already been extensively discussed elsewhere [11]. As a term of comparison, it is worth mentioning that the fertilization rate and the implantation rate for r-ICSI embryos at the cleavage stage are below the competency values proposed by the ESHRE Consensus for ICSI cycles (60 and 25%, respectively) [38] and that the overall success rate is in the range of ‘futility’ or ‘very poor prognosis’ according to the Ethics Committee of the American Society for Reproductive Medicine [39]. Conversely, results deriving from cryopreservation of embryos obtainable by r-ICSI deserve some attention. Indeed, according to the present results, the change in the procedure allows ameliorate the clinical pregnancy rate for embryo transfer with an OR = 4.7 (95% CI: 2.6–8.6) and the implantation rate with an OR = 3.3 (95%CI: 2.0–5.6). Undoubtedly, it has to be recognized that only five studies produced data on r-ICSI coupled with frozen embryo transfer and all of them were retrospective observational studies. No randomized clinical trial is currently available. However, all the studies consistently reported acceptable success rates and the demonstration of an effect size greater than 3 or 4, as for implantation and clinical pregnancy rates, respectively, may be considered worthwhile, taking into account that observational studies are often not able to assess weak associations [40]. Even considering the lower limits of confidence intervals and therefore the statistical variability linked to the characteristics of the studies, results remain of interest with clinical pregnancy rates per embryo transfer of about 30%. Nevertheless, given the retrospective nature of the studies, we cannot exclude the presence of possible selection biases and residual confounding factors that may have led to incorrect interpretation of causal associations.

Similar considerations may be applied to the three studies reporting results of the comparison between fresh and frozen embryo transfers after r-ICSI. On the other hand, it has to be noted that, in these studies, transfers with cryopreserved embryos were carried out with residual embryos following a fresh embryo transfer from the same r-ICSI cycles. Therefore, even though the experimental design is not based on a randomization, it is still an intra-patient model of some clinical interest.

Another possible limit of the studies considered is that they might have included a very selected cohort of patients and/or embryos. Indeed, it is possible that unsuccessful events were not published and that patients who achieved pregnancies with frozen r-ICSI cycles may be overrepresented. Similarly, although speculative, embryos that succeeded in implantation might have derived from a very selected cohort of oocytes with exceptionally high quality and developmental potential therefore only marginally affected by in vitro ageing. There are at least three relevant observations to consider in this regard. First, embryos judged to have the highest probability of implantation are generally transferred in fresh cycles and it is therefore plausible that the cryopreserved supernumerary embryos were not the top quality embryos of the reported cycle cohort. Despite this, they showed a higher implantation potential compared to the fresh counterparts. Second, based on studies with elective cryopreservation of embryos [36, 37], we can estimate in higher than 50% the proportion of women who actually succeeded in obtaining viable embryos for cryopreservation following r-ICSI. This data could have probably been influenced by the in vitro selection of embryos achievable through the culture up to the blastocyst stage; in fact, it has been reported that frozen r-ICSI embryos transferred at the blastocyst stage have a statistically significant higher implantation potential compared to the cleavage stage (41% versus 12%, respectively) [37]. Third, in the included studies, age and other variables as potential confounding factors have been controlled by intra-patient comparisons and should not have strongly impacted.

The available data are also limited by the lack of relevant clinical information since obstetric and perinatal findings were often not reported. Less than 180 births have been described so far deriving from both fresh and frozen cycles using r-ICSI; two miscarriages due to malformations and no relevant health problems in newborns with the exception of one case of microtia were reported. Therefore, although the cohort of babies born from this procedure is limited, present results do not suggest an increase of adverse outcomes following its application, including malformation rates.

Collectively, even considering the reported limits of the considered studies, the present findings highlight the consistent improvement in the success rate using frozen-thawed embryo transfer after late r-ICSI cycles.

This observation has important implications for clinical embryologists. The opportunity to rely on a rescue procedure with satisfactory chances of success could entice the operators to a greater use of the conventional IVF technique. An excessive use of ICSI, aiming at preventing cases of TFF, is thought to have negative consequences both on the overall probability of pregnancy and on the safety of the procedures with higher costs and increased laboratory workload. According to the results of a recent systematic review [6], TFF risk is significantly increased after conventional IVF insemination compared to ICSI (relative risk = 2.63, 95%CI: 1.29–5.35) in couples with non-male factor infertility; on the contrary, overall fertilization rates are not significantly improved with the use of ICSI and clinical pregnancy rates are even higher using conventional IVF. Similarly, a previous Cochrane review confirmed that conventional IVF gives better fertilization results than ICSI in couples with male factor subfertility and also suggested that pregnancy, miscarriage or live-birth rates after conventional IVF and ICSI are comparable for couple with non-male subfertility. If anything, ICSI does not improve the success rate in these couples [41]. Even if results from a recent meta-analysis favour the use of ICSI to increase fertilization rates and decrease the risk of TFF in couples with well defined unexplained infertility, no data on the impact on clinical pregnancy and live birth rates have been provided in this publication [8]. A recent randomised clinical trial failed to demonstrate an advantage of ICSI compared to conventional IVF in couples without male factor indication in terms of total fertilization failure, live birth and implantation rates [7]. Since age has been correlated with zona pellucida thickening, ICSI has been proposed for improving ART outcomes in older patients [42]; however, a recent prospective randomized controlled trial comparing conventional insemination versus ICSI on sibling oocytes in advanced maternal age patients showed similar fertilization rate, average number of cleavage stage and average top-quality embryos between the two groups (9). Given this scenario, it is essential to illustrate any beneficial role of r-ICSI in order to increase the confidence of embryologists in proceeding with conventional IVF.

Late r-ICSI can be easily implemented in ART laboratories as it can be carried out the day after oocyte retrieval and it is not difficult to fit it timely in the context of the laboratory process while this is often the case for early r-ICSI. Rescue ICSI performed as early as 6 h after in vitro insemination has been similarly proposed as an interesting treatment option to avoid a complete failure of conventional IVF [12, 13]. Overall, according to the review by Beck-Fruchter et al., a pregnancy rate of 44% can be achieved following the application of this procedure in cases of TFF [11]. Despite these encouraging results, early r-ICSI is still sporadically used and generally in laboratories located in China. The underlying plausible reason for this unpopularity relies on the organization problems that may arise in implementing this procedure in the context of the routine activities of standard IVF laboratories. It is therefore possible that late r-ICSI may find a greater consensus among operators if it proves to be equally efficient, even though it entails the need to apply the elective cryopreservation of the embryos. Cryopreservation procedures are currently well integrated among IVF laboratory treatments and can be routinely organized without very strict time requirements. Elective embryo freezing also allows conducting genetic testing in order to verify whether there is a concrete risk of genetic anomalies linked to the extension of the culture time between oocyte retrieval and insemination, as suggested in very preliminary case-reports [27, 29].

Of utmost interest, benefits of cryopreservation and transfer in subsequent cycles may explain the discrepancy between results derived from fresh or frozen/thawed embryo transfers. Indeed, some degree of asynchrony between embryo developmental stage and endometrial receptivity window may occur following fresh transfer of cleavage stage embryos derived from late r-ICSI [43]. Most of the reports indeed described results from transferring embryos at the cleavage stage [11]. Although endometrial receptivity is thought to have an extraordinary plasticity so that embryos could implant regardless of their precise phase of development (e.g., a cleavage-stage embryo could implant in an endometrium theoretically set to receive a blastocyst), we cannot exclude that small perturbations at the opening of the window of implantation may have a detrimental role.

Some critical variables may potentially affect the efficacy of late r-ICSI but, unfortunately, data are currently poorly available in this context. Among those variables, it is worth citing the following: 1) the use of 1-day old or freshly prepared spermatozoa; only one study compared the two types of ejaculate, suggesting that fresh spermatozoa are associated with higher fertilization rates. In all other reports, sperm used was collected on the day before. Since sperm quality and aging could explain, at least in part, observed fertilization rates which were found to be lower compared to standard ICSI cycles, this issue remains to be clarified through reliable information on the genetic and metabolic quality of spermatozoa after a 24-h incubation; 2) the rate of immature or nearly mature oocytes available at the time of conventional IVF. It is well known that the evaluation of nuclear maturity of oocytes still in their cumulus cells can be demanding and that metaphase I oocytes could benefit from in vitro culture until the day after retrieval in order to gain competence to undergo fertilization. For this reason, we cannot exclude that some positive results of late r-ICSI may be due to oocyte maturation rather than to the fertilization technique; 3) timing of oocyte retrieval after ovulation triggering and exposure of oocytes to sperm cells during conventional IVF insemination; both these aspects may in fact influence the rate of mature oocytes during the insemination window; 4) the specific freezing procedure. The vast majority of results using r-ICSI coupled with embryo freezing were obtained using the slow freezing procedure. Since vitrification is acquiring increasing popularity worldwide as a more efficient technique [44], we may assume that, in the near future, r-ICSI data will be positively influenced by the employment of vitrification. Finally, it has to be mentioned that some laboratories prefer to extend the culture of apparently unfertilized oocytes to the next day, in order not to discard viable embryos deriving from miscategorized zygotes. The employment of r-ICSI should not imply some changes in this approach since, at 24 h after insemination, some evidence of fertilization should already be present. The use of time-lapse may represent a valid option [45].

In conclusion, the results of this review support the idea that r-ICSI coupled with frozen embryo transfer may represent an efficient procedure for couples experiencing TFF following conventional IVF cycles. The strategy of embryo cryopreservation seems to overcome most of the technical and biological issues associated with a fresh transfer after late r-ICSI. Data derived from embryo vitrification instead of slow freezing will provide a definitive answer on this topic.

## Supplementary Information

**Additional file 1.** JBI critical appraisal checklist for analytical cross sectional studies. The evaluation of included studies made according to the Joanna Briggs Institute.

**Additional file 2.** Fertilization rate in late r-ICSI cycles. Effect size of fertilization rate (2 pronuclei) in included studies.

## Data Availability

The datasets used and/or analysed during the current study are available from the corresponding author on reasonable request.

## Abbreviations

ART: Assisted reproductive technologies
ICSI: Intracytoplasmic sperm injection
IVF: In-vitro fertilization
OR: Odds Ratio
PFF: Partial fertilization failure
r-ICSI: rescue-ICSI
TFF: Total fertilization failure
