# Supplementary figures and images for: Should rescue ICSI be re-evaluated considering the deferred transfer of cryopreserved embryos in in-vitro fertilization cycles? A systematic review and meta-analysis
Source: Reprod Biol Endocrinol. 2021 Aug 4;19:121. doi: 10.1186/s12958-021-00784-3 (PMC8336078; doi:10.1186/s12958-021-00784-3)

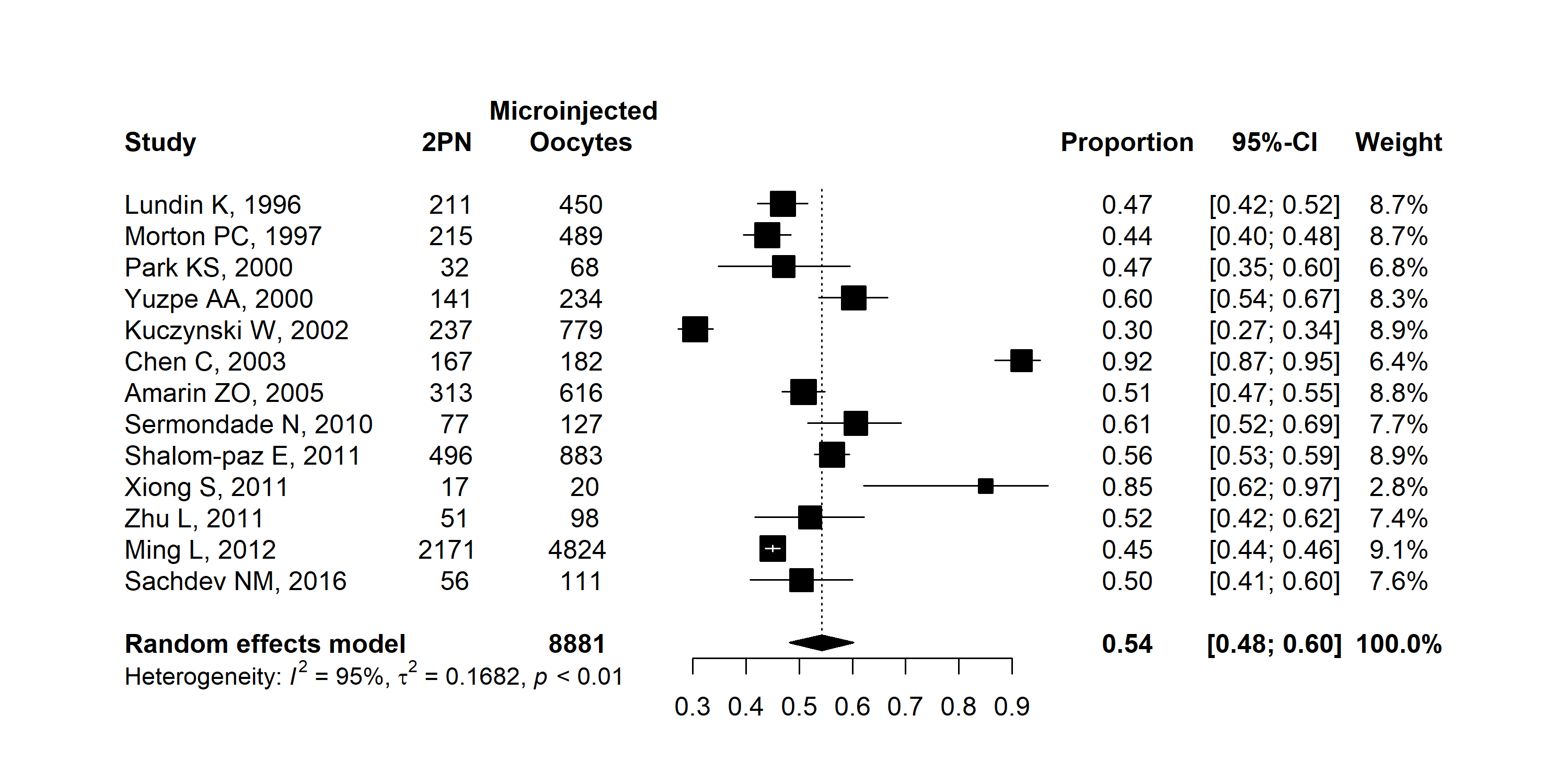

Supplement: Supplementary file 2 — Additional file 2. Fertilization rate in late r-ICSI cycles. Effect size of fertilization rate (2 pronuclei) in included studies. [file 12958_2021_784_MOESM2_ESM.tiff]
